# A prospective cohort study comparing efficacy of 1 dose of quadrivalent human papillomavirus vaccine to 2 and 3 doses at an average follow up of 12 years postvaccination

**DOI:** 10.1093/jncimonographs/lgae042

**Published:** 2024-11-12

**Authors:** Sylla G Malvi, Pulikkottil O Esmy, Richard Muwonge, Smita Joshi, Usha Rani Reddy Poli, Eric Lucas, Yogesh Verma, Pesona Grace Lucksom, Anand Shah, Bijal Patel, Eric Zomawia, Sharmila Pimple, Kasturi Jayant, Sanjay Hingmire, Aruna Chiwate, Uma Divate, Shachi Vashist, Gauravi Mishra, Radhika Jadhav, Maqsood Siddiqi, Catherine Sauvaget, Subha Sankaran, Thiraviam Pillai Rameshwari Ammal Kannan, Surendra S Shastri, M Radhakrishna Pillai, Devasena Anantharaman, Neerja Bhatla, Rengaswamy Sankaranarayanan, Partha Basu

**Affiliations:** Tata Memorial Centre Rural Cancer Project, Nargis Dutt Memorial Cancer Hospital, Barshi, Solapur, India; Department of Radiation Oncology, Christian Fellowship Community Health Centre, Dindigul, India; Early Detection, Prevention and Infections Branch, International Agency for Research on Cancer, Lyon, France; Jehangir Clinical Development Centre, Jehangir Hospital Premises, Pune, India; Public Health Foundation of India, Indian Institute of Public Health – Hyderabad, Hyderabad, India; Early Detection, Prevention and Infections Branch, International Agency for Research on Cancer, Lyon, France; Sikkim Manipal Institute of Medical Sciences, Sikkim Manipal University, Gangtok, Sikkim, India; Sikkim Manipal Institute of Medical Sciences, Sikkim Manipal University, Gangtok, Sikkim, India; Gujarat Cancer & Research Institute, M.P. Shah Cancer Hospital, Civil Hospital Campus, Asarwa, Ahmedabad, India; Gujarat Cancer & Research Institute, M.P. Shah Cancer Hospital, Civil Hospital Campus, Asarwa, Ahmedabad, India; Civil Hospital, Aizawl, Mizoram, India; Department of Preventive Oncology, Centre for Cancer Epidemiology, Homi Bhabha National Institute, Tata Memorial Centre, Mumbai, India; Tata Memorial Centre Rural Cancer Project, Nargis Dutt Memorial Cancer Hospital, Barshi, Solapur, India; Tata Memorial Centre Rural Cancer Project, Nargis Dutt Memorial Cancer Hospital, Barshi, Solapur, India; Tata Memorial Centre Rural Cancer Project, Nargis Dutt Memorial Cancer Hospital, Barshi, Solapur, India; Jehangir Clinical Development Centre, Jehangir Hospital Premises, Pune, India; Department of Obstetrics and Gynaecology, All India Institute of Medical Sciences, New Delhi, India; Department of Preventive Oncology, Centre for Cancer Epidemiology, Homi Bhabha National Institute, Tata Memorial Centre, Mumbai, India; Jehangir Clinical Development Centre, Jehangir Hospital Premises, Pune, India; Cancer Foundation of India, Kolkata, West Bengal, India; Early Detection, Prevention and Infections Branch, International Agency for Research on Cancer, Lyon, France; Cancer Research Division, Rajiv Gandhi Centre for Biotechnology, Thiruvananthapuram, Kerala, India; Cancer Research Division, Rajiv Gandhi Centre for Biotechnology, Thiruvananthapuram, Kerala, India; Department of Health Disparities Research, Division of Cancer Prevention and Population Sciences, University of Texas, Houston, TX, USA; Cancer Research Division, Rajiv Gandhi Centre for Biotechnology, Thiruvananthapuram, Kerala, India; Cancer Research Division, Rajiv Gandhi Centre for Biotechnology, Thiruvananthapuram, Kerala, India; Department of Obstetrics and Gynaecology, All India Institute of Medical Sciences, New Delhi, India; Karkinos Healthcare, Kerala Operations, Ernakulam, India; Early Detection, Prevention and Infections Branch, International Agency for Research on Cancer, Lyon, France

## Abstract

**Background:**

While recommending a human papillomavirus (HPV) single-dose vaccination schedule in 2022, the World Health Organization highlighted the need for long-term follow-up studies to monitor waning of protection. We report on vaccine efficacy against HPV infections in 1-, 2-, and 3-dose schedules and protection against cervical precancers at a median follow-up of 12 years postvaccination.

**Methods:**

This randomized multicenter study in India was originally designed to vaccinate unmarried girls aged 10-18 years with either 2 or 3 doses of quadrivalent HPV vaccine. A ministerial decree to halt vaccination in trials resulted in the creation of cohorts receiving different doses, including just a single dose. Cohorts were assessed for incident and persistent infections by genotyping cervical samples collected yearly for 4 consecutive years after participants were married. Cervical screening with an HPV test was initiated at age 25 years for married participants. Age- and site-matched unvaccinated married women were recruited to be compared with vaccinated cohorts. Vaccine efficacy was assessed using proportional incidence ratios.

**Results:**

The number of participants in the 1-, 2- (at 0 and 6 months), and 3-dose cohorts was 4949, 4980, and 4348, respectively. Of the recipients, 71%-82% in the different cohorts were eligible to provide samples for genotyping. Vaccine efficacy against persistent HPV 16 and 18 infection was 92.0% (95% confidence interval [CI] = 87.0% to 95.0%) in 3022 recipients of the single dose; and comparable with that observed in the 2-dose arm (94.8%, 95% CI = 90.0% to 97.3%) and the 3-dose arm (95.3%, 95% CI = 90.9% to 97.5%). No high-grade precancer associated with HPV 16 and 18 was detected among vaccinated participants compared with 8 precancers detected among the unvaccinated women.

**Conclusion:**

This observational cohort study has established that a single dose of HPV vaccine provides high protective efficacy against persistent HPV 16 and 18 infections and associated neoplasia 15 years postvaccination.

The first vaccine against human papillomavirus (HPV) was authorized for sale nearly 2 decades ago. Randomized controlled trials evaluating 3 doses of the vaccine demonstrated high efficacy in preventing high-grade cervical intraepithelial neoplasia (CIN) associated with vaccine-targeted HPV types (1). Protection durability was subsequently demonstrated by following up on trial participants for 14 years postvaccination; vaccine efficacy was 100% against HPV16- and 18-related high-grade CIN (2). The World Health Organization (WHO) switched from a 3-dose recommendation to a 2-dose schedule in 2014 for girls aged 9-13 years (3). Evidence derived from data linkage studies from countries that introduced the vaccine early in national immunization programs supported the robust effectiveness of 2 or 3 doses of the vaccine to prevent cervical cancers (4).

Despite high efficacy and safety of HPV vaccines, several countries have yet to introduce the vaccine into national immunization programs (141 have introduced to date), and only 21% of girls had received at least 1 dose up to 2022 (5). The primary target population (girls aged 9-14 years) in several low- and middle-income countries—with a high burden of cervical cancer—do not have access to the vaccine. Major barriers are the high cost of administering 2 doses (price per dose ranging from US$10 to US$64 for self-procuring middle-income countries) and logistic complexities of reaching out to the adolescent girls and finding them for the second dose, as well as vaccine shortages (6). It is therefore not surprising that vaccine coverage in 2022 for the last dose in females by 15 years of age was a meager 2% in Southeast Asia and only 19% in Africa (7).

The WHO position paper on HPV vaccines (2022 update) recommends a single dose for girls and boys aged 9-20 years as an off-label option, which would considerably improve affordability and programmatic feasibility as well as efficiency by overcoming these barriers. Accepting that the available immunogenicity and efficacy data supporting a single dose was sufficiently compelling, WHO experts also highlighted the need for long-term follow-up studies to monitor waning protection over time (8).

The International Agency for Research on Cancer–WHO (IARC/WHO), France, initiated a multicentric randomized controlled trial in India in 2009 to compare vaccine efficacy of 2 vs 3 doses of quadrivalent vaccine (Gardasil, Merck & Co., Inc., Rahway, NJ, USA). Vaccination was abruptly halted in April 2010 because of a ministerial decree, which resulted in the creation of cohorts of females receiving different doses, including only a single dose. In the present manuscript, we report comparative efficacies against incident and persistent HPV infections and cervical precancers and cancers among the 1-, 2-, and 3-dose arms 15 years after start of vaccination.

## Methods

**Figure 1.**
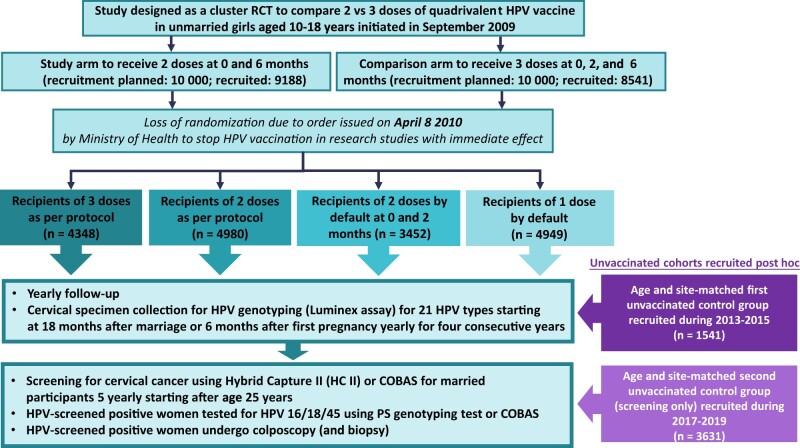
Study flowchart. HPV = human papillomavirus; RCT = randomized controlled trial.

The study design flowchart is described in Figure 1. Recruitment in the cluster randomized controlled trial started in September 2009 at 9 locations across 7 states of India. The original objective of this randomized controlled trials was to compare the efficacy of 2 doses of Gardasil administered at days 1 and 180 to the same vaccine being administered with 3 doses at days 1, 60, and 180. The protocol specified recruitment of 20 000 unmarried girls aged 10-18 years who were not vaccinated against HPV from 188 geographical clusters. The clusters were randomly assigned in a 1:1 ratio to the 2- or 3-dose arms. The government of India issued a notification on April 8, 2010, to stop HPV vaccination in all research studies following a few nonvaccine-related deaths reported in another trial. On the date of vaccination suspension, 17 729 females (88.6% of the recruitment target) were already randomly assigned and had received at least 1 dose. Early termination of vaccination resulted in the spontaneous formation of 4 dose cohorts with participants receiving either 3 or 2 doses as per protocol, 2 doses by default at days 1 and 60, or 1 dose by default. Cohorts were followed up yearly and assessed for incident and persistent HPV infections and cervical screening outcomes. Details of the follow-up procedures were reported in our earlier publications and are briefly described below (9,10).

Participants were contacted once a year by study staff to check their general health status and update their contact details and marital status. Participants were invited to a clinic to provide cervical samples for HPV genotyping 18 months after marriage or 6 months after first childbirth, whichever was earlier. Samples were not collected earlier because in the predominantly rural and conservative society of India, obtaining information about sexual practices or performing any gynecological examinations before marriage is not socioculturally acceptable. Cervical samples were collected yearly for 4 consecutive years by care providers in PreservCyt (Hologic, Marlborough, MA, USA) medium. A fifth sample was collected from participants who tested positive for HPV infection in the last sample. Samples were analyzed with a type-specific E7 polymerase chain reaction multiplex assay (Luminex, Austin, TX, USA) to detect 19 high-risk (or probable high-risk) types (HPV 16, 18, 26, 31, 33, 35, 39, 45, 51, 52, 53, 56, 58, 59, 66, 68, 70, 73, 82) and 2 low-risk types (HPV 6 and 11).

Cervical samples were collected at the first round of screening from married participants aged 25 years and older. These samples were analyzed with the Hybrid Capture-II (HC-II) test (Qiagen, Gaithersburg, MD, USA) that detects the 13 most oncogenic HPV types. Positive samples using HC-II were further tested with the Digene HPV genotyping PS test (Qiagen, Gaithersburg, MD, USA) for qualitative detection of HPV types 16, 18, and 45. Since August 2023, samples are analyzed using the COBAS-6800 (Roche Diagnostics, Rotkreuz, Switzerland) platform. COBAS-6800 detects HPV16 and 18 individually, along with simultaneous, pooled detection of 12 other high-risk HPV types.

Participants positive for screening tests were invited for colposcopy, and the other participants were advised to return for screening in 5 years. Cervical punch biopsies were taken if the Swede score on colposcopy was at least 5. Detected CIN 3 or cancer were appropriately managed and excluded from the study. Participants with Swede score less than 5 or no more than CIN 2 on histopathology were advised to follow up with an HPV test 1 year later. If the follow-up test was positive, colposcopy was performed and biopsies were taken from any visible lesion or randomly from 2 quadrants with no visible lesions.

A cohort of unvaccinated married women (n = 1486) matched by age and study site to the vaccinated women was recruited from 2013 to 2015. Cervical samples for HPV genotyping were collected yearly for 4 consecutive years and were analyzed using Luminex assay, as was done for the vaccinated participants. Unvaccinated participants were also screened for cervical cancer starting at age 25 years following the same protocol for vaccinated participants. A second unvaccinated cohort of married women (n = 3690) was recruited from 2017 to 2019 to increase the number of unvaccinated women for assessment of the CIN 2 or worse endpoints. The second group was age and site matched to the screening-eligible vaccinated women and only screened for cervical cancer (screening-only cohort) following the protocol. No samples were collected from the second group for Luminex genotyping.

Laboratory staff performing HPV tests, colposcopists, and histopathologists were blinded to participants’ vaccination status. The study was approved by ethics committees of IARC and the collaborating institutions. Written informed consent was obtained from a parent at enrollment. Participants signed an assent form. As participants turned 18 years of age, they signed an informed consent form to participate in follow-up. This trial is registered with International Standard Randomised Controlled Trial Number (ISRCTN), ISRCTN98283094, and ClinicalTrials.gov, NCT00923702.

### Study outcomes

The primary outcome assessed in this analysis was persistent HPV 16 and/or 18 infection. We looked at incident HPV 16 and 18 infections, and HPV 16- and 18-related CIN 2 or worse lesions as secondary outcomes and, as exploratory outcomes, incident and persistent infections from HPV 6 and 11; HPV 31, 33, and 45; oncogenic high-risk HPV 16, 18, 31, 33, 35, 39, 45, 51, 52, 56, 58, 59, 66, and 68 included in the screening tests; and any HPV. For HPV incidence and persistence outcomes, the dates of the first and last cervical cell sample collections were used as event start and end dates, respectively, in the vaccinated and unvaccinated groups. As for the CIN outcome, the event started on the date of the first round of screening. Follow-up time for HPV incidence and persistence outcomes was estimated for the vaccinated groups from the date of first vaccine dose to the date of the last cervical cell sample collection.

An HPV infection was defined as incident if detected in any sample and as persistent if detected in 2 consecutive samples collected at least 10 months apart. Each participant was counted only once in the analysis. Both HPV outcomes were assessed using the Luminex assay.

### Statistical analysis

The profile of participants providing samples for HPV genotyping and attending cervical cancer screening and the estimation of incident and persistent infections was presented as proportions and as a median together with their interquartile ranges (more details in the Supplementary Material, available online).

Vaccine efficacy against the study outcomes was assessed using proportional incidence ratios as explained by P Sasieni (additional details in the Supplementary Material, available online) (11). The difference between vaccine efficacy estimates of the 1 dose and the other dose scheduels (2-dose or 3-dose) was calculated using methods developed by Sampson and Gail (12). If the 95% confidence interval (CI) of the difference included zero, the vaccine efficacy of the two groups compared was deemed not to be statistically significant. Efficacy of the 2-dose default group is not presented here as the schedule would be of no public health importance.

## Results

The number and profile of the participants included in the analyses of incident and persistent infections by vaccine dose group are described in Table 1. Compliance from the eligible participants to provide cervical samples at baseline and at least 2 samples later was high (approximately 90%). Median interval between first dose vaccination and last sample collection was approximately 12 years in each dose group. Median age at first sample collection, median interval between marriage and first sample collection, and median gap between consecutive sample collections were comparable between different dose groups and unvaccinated women.

**Table 1.** Profile of participants providing cervical samples for analysis of HPV genotypes and cervical cancer screening

Profile item	Vaccine dose schedule received				Unvaccinated group
	3 doses (days 1, 60, and ≥180)	2 doses (days 1 and ≥180)	2 doses default (days 1 and 60)	1 dose	
Women recruited, No.	4348	4980	3452	4949	1486
Women eligible^a^ for sample collection for HPV genotyping, No.	3068	3335	2834	3903	1486
%	70.6	67.0	82.1	78.9	100.0
Profile of participants included in the HPV genotyping analysis (September 2009 to March 2023)					
Women with at least 1 sample analyzed, No.	2739	2983	2584	3602	1486
%	89.3	89.4	91.2	92.3	100.0
Women eligible for the second sample collection among those whose first sample was analyzed, No.	2475	2665	2453	3394	1486
%	90.4	89.3	94.9	94.2	100.0
Women with at least a second sample analyzed, No.	2294	2456	2285	3140	1276
%	92.7	92.2	93.2	92.5	85.9
Median age at first sample collection, y	22.0	22.0	21.0	21.0	21.0
IQR	20.0-24.0	20.0-24.0	19.0-23.0	19.0-23.0	20.0-21.0
Range	14.0-31.0	14.0-31.0	14.0-31.0	13.0-31.0	18.0-23.0
Median time between first vaccination dose and last sample collection, y	12.3	12.3	11.6	11.7	
IQR	10.0-13.5	10.0-13.5	9.3-12.9	9.5-13.0	
Range	2.7-14.5	2.8-14.5	2.0-14.4	2.2-14.4	
Median time between marriage and first cervical cell sample collection, y	2.0	1.9	2.0	2.1	2.8
IQR	1.6-2.6	1.6-2.7	1.5-2.8	1.6-3.1	1.9-4.2
Range	−4.2-12.6	−8.5-10.7	0.0-10.1	−4.4-12.4	−2.1-12.8
Median time between consecutive sample collections, y	1.3	1.3	1.4	1.3	1.4
IQR	1.1-1.8	1.1-1.7	1.1-1.9	1.1-1.8	1.1-1.8
Range	0.5-10.3	0.5-10.6	0.8-8.8	0.7-9.8	0.8-10.3
Profile of participants included in cervical cancer screening (September 2009 to March 2024)					
Women eligible^b^ for screening, No.	2905	3163	2637	3609	5176^c^
%	66.8	63.5	76.4	72.9	
Women screened at least once, No.	2436	2635	2200	3005	4742
%	83.9	83.3	83.4	83.3	91.6
Screen negative women undergoing second round of screening, No.	392	442	449	661	2793
%	16.1	16.8	20.4	22.0	58.9
Median age at first screen, y	25.0	25.0	25.0	25.0	26.0
IQR	25.0-26.0	25.0-26.0	25.0-26.0	25.0-26.0	25.0-27.0
Range	20.0-32.0	21.0-33.0	22.0-32.0	21.0-32.0	19.0-35.0
Median time between first vaccination dose and first screen, y	11.8	11.6	11.1	11.1	
IQR	9.5-12.9	9.4-12.7	9.1-12.5	9.0-12.4	
Range	6.5-14.5	6.5-14.4	6.7-14.4	6.6-14.4	

a Eligible 18 months after marriage or 6 months after first childbirth, whichever is first. HPV = human papillomavirus; IQR = interquartile range.

b Eligible on turning 25 years of age and being married.

c Includes 3690 unvaccinated women recruited post hoc as controls to screen eligible women.

Outcomes for incident and persistent infections from different genotypes and their combinations by dose cohorts are shown in Table 2. Incident HPV 16 and 18 infection was detected in 3.1% (95% CI = 2.6% to 3.85) of the 3602 single-dose recipients included in the analysis. The proportion was similar to that for 2- or 3-dose recipients and substantially lower compared with the unvaccinated women (9.8%, 95% CI = 8.4% to 11.5%). The proportion of 1-, 2-, and 3-dose recipients having incident infections from vaccine-targeted HPV types (HPV 16, 18, 6, 11) and cross-protective types (HPV 31, 33, 45) was comparable. A significantly higher proportion of unvaccinated women had incident infections from nonvaccine-targeted HPV types (also excluding HPV 31, 33, 45) (29.3%, 95% CI = 27.0% to 31.7%) compared with those receiving any number of doses (17.8%, 95% CI = 17.1% to 18.5%; *P* < .001). This observation means that the unvaccinated participants, though matched by age and study sites, were exposed to a higher risk of being infected with HPV compared with the vaccinated women.

**Table 2.** Analysis of incident HPV infections and persistent HPV infections by number of doses received^a^

Type of HPV infection and dose received	Incident HPV infections in participants with at least 1 sample tested			Incident persistent HPV infections in participants with at least 2 samples tested		
	Women assessed, No.	Women with incident infection, No.	Proportion of incidence, % (95% CI)	Women assessed, No.	Women with persistent infections, No.	Proportion of persistence, % (95% CI)
Women with samples tested	13394	493		10 981	47	
HPV 16 and 18 infections						
Unvaccinated group	1486	146	9.8 (8.4–11.5)	1273	35	2.7 (1.9–3.8)
Vaccinated group	11 908	347	2.9 (2.6–3.2)	9708	12	0.1 (0.1–0.2)
1 dose	3602	113	3.1 (2.6–3.8)	3022	4	0.1 (0.0–0.3)
3 doses (days 1, 60, and ≥180)	2739	77	2.8 (2.2–3.5)	2172	2	0.1 (0.0–0.3)
2 doses (days 1 and ≥180)	2983	73	2.4 (1.9–3.1)	2311	2	0.1 (0.0–0.3)
2 doses (days 1 and 60)	2584	84	3.3 (2.6–4.0)	2203	4	0.2 (0.0–0.5)
HPV 16 infections						
Unvaccinated group	1486	101	6.8 (5.6–8.2)	1273	25	2.0 (1.3–2.9)
Vaccinated group	11 908	225	1.9 (1.7–2.2)	9708	2	0.0 (0.0–0.1)
1 dose	3602	68	1.9 (1.5–2.4)	3022	1	0.0 (0.0–0.2)
3 doses (days 1, 60, and ≥180)	2739	55	2.0 (1.5–2.6)	2172	0	0.0 (0.0–0.2)
2 doses (days 1 and ≥180)	2983	42	1.4 (1.0–1.9)	2311	0	0.0 (0.0–0.2)
2 doses (days 1 and 60)	2584	60	2.3 (1.8–3.0)	2203	1	0.0 (0.0–0.3)
HPV 18 infections						
Unvaccinated group	1486	56	3.8 (2.9–4.9)	1273	11	0.9 (0.4–1.5)
Vaccinated group	11 908	136	1.1 (1.0–1.3)	9708	10	0.1 (0.0–0.2)
1 dose	3602	50	1.4 (1.0–1.8)	3022	3	0.1 (0.0–0.3)
3 doses (days 1, 60, and ≥180)	2739	27	1.0 (0.7–0.0)	2172	2	0.1 (0.0–0.3)
2 doses (days 1 and ≥180)	2983	31	1.0 (0.7–1.5)	2311	2	0.1 (0.0–0.3)
2 doses (days 1 and 60)	2584	28	1.1 (0.7–1.6)	2203	3	0.1 (0.0–0.4)
HPV 6 and 11 infections						
Unvaccinated group	1486	82	5.5 (4.4–6.8)	1273	3	0.2 (0.0–0.7)
Vaccinated group	11 908	638	5.4 (5.0–5.8)	9708	13	0.1 (0.1–0.2)
1 dose	3602	186	5.2 (4.5–5.9)	3022	3	0.1 (0.0–0.3)
3 doses (days 1, 60, and ≥180)	2739	155	5.7 (4.8–6.6)	2172	4	0.2 (0.1–0.5)
2 doses (days 1 and ≥180)	2983	174	5.8 (5.0–6.7)	2311	3	0.1 (0.0–0.4)
2 doses (days 1 and 60)	2584	123	4.8 (4.0–5.7)	2203	3	0.1 (0.0–0.4)
Vaccine-targeted HPV (16, 18, 6, and 11) infections						
Unvaccinated group	1486	217	14.6 (12.8–16.5)	1273	38	3.0 (2.1–4.1)
Vaccinated group	11 908	941	7.9 (7.4–8.4)	9708	25	0.3 (0.2–0.4)
1 dose	3602	288	8.0 (7.1–8.9)	3022	7	0.2 (0.1–0.5)
3 doses (days 1, 60, and ≥180)	2739	218	8.0 (7.0–9.0)	2172	6	0.3 (0.1–0.6)
2 doses (days 1 and ≥180)	2983	236	7.9 (7.0–8.9)	2311	5	0.2 (0.1–0.5)
2 doses (days 1 and 60)	2584	199	7.7 (6.7–8.8)	2203	7	0.3 (0.1–0.7)
HPV 31 and 33 infections						
Unvaccinated group	1486	137	9.2 (7.8–10.8)	1273	13	1.0 (0.5–1.7)
Vaccinated group	11 908	398	3.3 (3.0–3.7)	9708	39	0.4 (0.3–0.5)
1 dose	3602	141	3.9 (3.3–4.6)	3022	15	0.5 (0.3–0.8)
3 doses (days 1, 60, and ≥180)	2739	92	3.4 (2.7–4.1)	2172	10	0.5 (0.2–0.8)
2 doses (days 1 and ≥180)	2983	99	3.3 (2.7–4.0)	2311	12	0.5 (0.3–0.9)
2 doses (days 1 and 60)	2584	66	2.6 (2.0–3.2)	2203	2	0.1 (0.0–0.3)
HPV 31, 33, and 45 infections						
Unvaccinated group	1486	157	10.6 (9.0–12.2)	1273	17	1.3 (0.8–2.1)
Vaccinated group	11 908	484	4.1 (3.7–4.4)	9708	54	0.6 (0.4–0.7)
1 dose	3602	173	4.8 (4.1–5.6)	3022	17	0.6 (0.3–1.2)
3 doses (days 1, 60 and ≥180)	2739	110	4.0 (3.3–4.8)	2172	16	0.7 (0.4–1.2)
2 doses (days 1 and ≥180)	2983	122	4.1 (3.4–4.9)	2311	18	0.8 (0.5–1.2)
2 doses (days 1 and 60)	2584	79	3.1 (2.4–3.8)	2203	3	0.1 (0.0–0.4)
Nonvaccine-targeted HPV infections excluding 31, 33 and 45						
Unvaccinated group	1486	435	29.3 (27.0–31.7)	1273	81	6.4 (5.1–7.8)
Vaccinated group	11 908	2118	17.8 (17.1–18.5)	9708	370	3.8 (3.4–4.2)
1 dose	3602	618	17.2 (15.9–18.4)	3022	115	3.8 (3.2–4.6)
3 doses (days 1, 60, and ≥180)	2739	550	20.1 (18.6–21.6)	2172	98	4.5 (3.7–5.5)
2 doses (days 1 and ≥180)	2983	579	19.4 (18.0–20.9)	2311	89	3.9 (3.1–4.7)
2 doses (days 1 and 60)	2584	371	14.4 (13.0–15.8)	2203	68	3.1 (2.4–3.9)
14 most oncogenic HPV (16, 18, 31, 33, 35, 39, 45, 51, 52, 56, 58, 59, 66, and 68) infections						
Unvaccinated group	1486	514	34.6 (32.2–37.1)	1273	100	7.9 (6.4–9.5)
Vaccinated group	11 908	2305	19.4 (18.7–20.1)	9708	350	3.6 (3.2–4.0)
1 dose	3602	688	19.1 (17.8–20.4)	3022	105	3.5 (2.9–4.2)
3 doses (days 1, 60 and ≥180)	2739	602	22.0 (20.4–23.6)	2172	97	4.5 (3.6–5.4)
2 doses (days 1 and ≥180)	2983	603	20.2 (18.8–21.7)	2311	87	3.8 (3.0–4.6)
2 doses (days 1 and 60)	2584	412	15.9 (14.6–17.4)	2203	61	2.8 (2.1–3.5)
Any HPV (16, 18, 6, 11, 26, 31, 33, 35, 39, 45, 51, 52, 53, 56, 58, 59, 66, 68, 70, 73, and 82) infection						
Unvaccinated group	1486	596	40.1 (37.6–42.7)	1273	114	9.0 (7.4–10.7)
Vaccinated group	11 908	3041	25.5 (24.8–26.3)	9708	424	4.4 (4.0–4.8)
1 dose	3602	912	25.3 (23.9–26.8)	3022	132	4.4 (3.7–5.2)
3 doses (days 1, 60, and ≥180)	2739	765	27.9 (26.3–29.7)	2172	111	5.1 (4.2–6.1)
2 doses (days 1 and ≥180)	2983	804	27.0 (25.4–28.6)	2311	105	4.5 (3.7–5.5)
2 doses (days 1 and 60)	2584	560	21.7 (20.1–23.3)	2203	76	3.4 (2.7–4.3)

a CI = confidence interval; HPV: human papillomavirus.

Based on analysis of at least 2 consecutive cervical samples, persistent HPV 16 and 18 infection could be assessed in 9708 vaccinated and 1273 unvaccinated women (Table 2). Only 1 (0.0%, 95% CI = 0.0% to 0.2%) persistent HPV 16 infection was detected among 3022 single-dose recipients included in the analysis. One persistent HPV 16 infection was also detected in the 2-dose default group (0.0%, 95% CI = 0.0% to 0.3%), but no persistent infections were found in the 2- or 3-dose groups. In comparison, the proportion of persistent HPV 16 infections detected in unvaccinated women was 2.0% (95% CI = 1.3% to 2.9%). The last 2 cervical samples taken from the single-dose recipients with persistent HPV 16 infections were HPV 16 negative.

HPV 18 persistent infections were detected in 2 participants (0.1%, 95% CI = 0.0% to 0.3%) in the 2- and 3-dose groups and in 3 participants in the 2 doses default (0.1%, 95% CI = 0.0% to 0.4%) and 1-dose (0.1%, 95% CI = 0.0% to 0.3%) groups. Persistent HPV 18 infection was detected in 0.9% (95% CI = 0.4% to 1.5%) of the unvaccinated women. The proportion of persistent HPV 16 and 18 infections was the same (0.1%, 95% CI = 0.0% to 0.3%) for 1, 2, or 3 doses and substantially lower among unvaccinated women (2.7%, 95% CI = 1.9% to 3.9%).

The number of persistent HPV 6 and 11 infections was very low in vaccinated (any dose) and unvaccinated women. The proportion of single-dose recipients with persistent HPV 31, 33, and 45 infections was substantially lower compared with the unvaccinated women (0.6%, 95% CI = 0.3% to 0.9%, vs 1.3%, 95% CI = 0.8% to 2.1%; *P =* .009).

Cervical screening was performed on 8012 vaccinated and 4734 unvaccinated women (Table 3). The 2-dose default group was excluded from this analysis. Screening test positivity was 7.9% in the unvaccinated and 5.5% in the vaccinated women (*P* <.001). The proportion was 4.6%, 5.6%, and 6.3% in 1-, 3-, and 2-dose recipients, respectively. Genotyping of the HPV-positive women using Digene PS or COBAS test revealed much lower prevalence of HPV 16 and 18 (individually or combined) in the vaccinated compared with the unvaccinated participants with no difference between the dose groups (Table 3). Only 1 HPV-16 infection (in combination with HPV 18) and 11 HPV-18 infections were detected among 2975 single-dose recipients.

**Table 3.** HPV screening test (Hybrid Capture II or COBAS 6800) positivity by HPV vaccine dose received

Vaccine dose received	Women screened, No.	High-risk HPV positivity on HC-II/COBAS^a^				
		Overall, No. (%)	HPV typing results available, No. (%)	HPV 16 alone, No. (%)	HPV 18 alone, No. (%)	HPV 16 and HPV 18, No. (%)
Women tested	12 746	813 (6.4)	789 (97.0)	63 (0.5)	70 (0.5)	11 (0.1)
Unvaccinated group	4734	374 (7.9)	367 (98.1)	62 (1.3)	35 (0.7)	7 (0.1)
Vaccinated group	8012	439 (5.5)	422 (96.1)	1 (0.0)	35 (0.4)	4 (0.0)
1 dose	2987	138 (4.6)	130 (94.2)	0 (0.0)	11 (0.4)	1 (0.0)
3 doses (days 1, 60, and ≥180)	2418	136 (5.6)	130 (95.6)	1 (0.0)	12 (0.5)	0 (0.0)
2 doses (days 1 and ≥180)	2607	165 (6.3)	162 (98.2)	0 (0.0)	12 (0.5)	3 (0.1)

a Screening test was switched from Hybrid Capture II (HC-II) to COBAS in September 2023; HC-II–positive women underwent Digene PS test (Qiagen, Gaithersburg, MD, USA) for HPV 16 and 18 genotyping, and genotyping information was included in the same run with COBAS 6800. HPV = human papillomavirus.

Further evaluation outcomes for the HPV-positive women by dose groups are described in Table 4. In the unvaccinated group, 79.7% (298 of 374) of HPV-positive women underwent colposcopy and 72.3% (319 of 439) in the vaccinated group. A total of 12 CIN 2 or CIN 3 lesions and 1 squamous cell carcinoma were detected in the unvaccinated women, while 4 CIN 2 or CIN 3 lesions and no cancers were detected in the vaccinated women. None of the CIN 2 or CIN 3 lesions in the vaccinated women was associated with HPV 16 or 18. These genotypes were detected in 8 CIN 2 or CIN 3 lesions in the unvaccinated women. Genotype information was unavailable for the cancer detected in the unvaccinated group.

**Table 4.** Final diagnosis among the HPV test positive women by vaccine dose received

Vaccine dose received	Women positive on HPV test, No.	Final diagnosis available among women positive on HC-II, No. (%)	CIN diagnosed				CIN diagnosed among women positive on HC-II for HPV types 16 and/or 18			
			CIN 1, No.	CIN 2, No.	CIN 3, No.	Invasive cancer, No.	CIN 1, No.	CIN 2, No.	CIN 3, No.	Invasive cancer, No.
Unvaccinated group	374	298 (79.7)	12	5	7	1	4	3	5	0
Vaccinated group	439	319 (72.7)	15	3	1	0	2	0	0	0
1 dose	138	108 (78.3)	4	0	1	0	0	0	0	0
3 doses (days 1, 60, and ≥180)	136	103 (75.7)	6	3	0	0	1	0	0	0
2 doses (days 1 and ≥180)	165	108 (65.5)	5	0	0	0	1	0	0	0

a The invasive cancer had no HPV genotyping results. CIN = cervical intraepithelial neoplasia; HC-II = hybrid capture 2; HPV = human papillomavirus.

Vaccine efficacy against incident HPV 16 and 18 infections was 45.5% (95% CI = 39.4% to 51.0%), 62.4% (95% CI = 57.4% to 66.9%), and 58.3% (95% CI = 52.8% to 63.1%) for the 1-, 2-, and 3-dose groups, respectively. More details about vaccine efficacy against incident HPV infections are provided in Table 5.

**Table 5.** Vaccine efficacy against incident HPV infections

Vaccine efficacy by HPV types	Unvaccinated	1 dose	2 doses (days 1 and ≥180)	3 doses (days 1, 60, and ≥180)
Women assessed, No.	1486	3602	2983	2739
Incident HPV 16 and/or 18 infections				
Observed events, No.	146	113	73	77
Crude attack rates, %	9.8	3.1	2.4	2.8
Vaccine efficacy,^a^ %		45.5	62.4	58.3
95% CI		(39.4 to 51.0)	(57.4 to 66.9)	(52.8 to 63.1)
Difference in vaccine efficacy				
Alternate dose, 1 dose, %			16.9	12.8
95% CI			(4.7 to 29.7)	(0.2 to 25.8)
Incident HPV 6 and/or 11 infections				
Observed events	82	186	174	155
Crude attack rates, %	5.5	5.2	5.8	5.7
Vaccine efficacy,^a^ %		−59.7	−59.4	−49.5
95% CI		(−79.0 to −42.4)	(−78.9 to −42.1)	(−68.2 to −32.9)
Difference in vaccine efficacy				
Alternate dose, 1 dose, %			0.2	10.2
95% CI			(−27.9 to 28.3)	(−17.8 to 38.7)
Incident HPV 16, 18, 6, and/or 11 infections				
Observed events	217	288	236	218
Crude attack rates, %	14.6	8.0	7.9	8.0
Vaccine efficacy,^a^ %		6.6	18.3	20.5
95% CI		(0.5 to 12.3)	(12.5 to 23.7)	(14.8 to 25.9)
Difference in vaccine efficacy				
Alternate dose, 1 dose, %			11.7	14.0
95% CI			(0.4 to 23.1)	(2.6 to 25.6)
Incident HPV types 31, 33, and/or 45 infections				
Observed events	157	173	122	110
Crude attack rates, %	10.6	4.8	4.1	4.0
Vaccine efficacy,^a^ %		22.4	41.6	44.6
95% CI		(15.2 to 29.0)	(35.4 to 47.2)	(38.5 to 50.1)
Difference in vaccine efficacy				
Alternate dose, 1 dose, %			19.2	22.1
95% CI			(5.7 to 33.2)	(8.5 to 36.3)
Any incident HPV infections				
Observed events	596	912	804	765
Crude attack rates, %	40.1	25.3	27.0	27.9
Vaccine efficacy,^a^ %		−7.7	−1.3	−1.5
95% CI		(−11.3 to −4.2)	(−4.7 to 1.9)	(−4.9 to 1.8)
Difference in vaccine efficacy				
Alternate dose, 1 dose, %			6.4	6.2
95% CI			(1.1 to 11.7)	(0.9 to 11.6)
Incident infection from high-risk HPV types (16, 18, 31, 33, 35, 39, 45, 51, 52, 56, 58, 59, 66, 68) included in the screening tests				
Observed events	514	688	603	602
Crude attack rates, %	34.6	19.1	20.2	22.0
Vaccine efficacy,^a^ %		5.8	11.9	7.4
95% CI		(3.7 to 7.8)	(10.1 to 13.6)	(5.3 to 9.4)
Difference in vaccine efficacy				
Alternate dose, 1 dose, %			6.1	1.6
95% CI			(−0.7 to 12.9)	(−5.3 to 8.4)
Incident HPV 16 infections				
Observed events	101	68	42	55
Crude attack rates, %	6.8	1.9	1.4	2.0
Vaccine efficacy,^a^ %		52.6	68.8	56.9
95% CI		(45.5 to 58.8)	(63.0 to 73.6)	(49.9 to 63.0)
Difference in vaccine efficacy				
Alternate dose, 1 dose, %			16.1	4.3
95% CI			(2.0 to 31.2)	(−11.2 to 19.9)
Incident HPV 18 infections				
Observed events	56	50	31	27
Crude attack rates, %	3.8	1.4	1.0	1.0
Vaccine efficacy,^a^ %		37.2	58.4	61.9
95% CI		(24.8 to 47.5)	(48.7 to 66.2)	(52.5 to 69.4)
Difference in vaccine efficacy				
Alternate dose, 1 dose, %			21.3	24.7
95% CI			(−0.8 to 46.2)	(2.6 to 49.8)
Incident HPV 31 infections				
Observed events	97	109	63	65
Crude attack rates, %	6.5	3.0	2.1	2.4
Vaccine efficacy,^a^ %		20.9	51.2	47.0
95% CI		(10.6 to 30.0)	(43.6 to 57.8)	(38.8 to 54.1)
Difference in vaccine efficacy				
Alternate dose, 1 dose, %			30.3	26.1
95% CI			(12.5 to 50.0)	(7.8 to 45.9)
Incident HPV types 33 infections				
Observed events	45	34	38	27
Crude attack rates, %	3.0	0.9	1.3	1.0
Vaccine efficacy,^a^ %		46.8	36.6	52.5
95% CI		(34.2 to 57.0)	(22.1 to 48.3)	(40.4 to 62.2)
Difference in vaccine efficacy				
Alternate dose, 1 dose, %			10.3	5.7
95% CI			(−39.1 to 16.3)	(−20.3 to 32.1)
Incident HPV types 45 infections				
Observed events	26	34	27	22
Crude attack rates, %	1.7	0.9	0.9	0.8
Vaccine efficacy,^a^ %		8.0	22.0	33.1
95% CI		(−17.6 to 28.0)	(−1.1 to 39.8)	(12.0 to 49.1)
Difference in vaccine efficacy				
Alternate dose, 1 dose, %			14.0	25.1
95% CI			(−29.9 to 61.4)	(−17.1 to 73.6)
Other incident nonvaccine-targeted HPV types excluding 31, 33, and 45 infections				
Observed events	435	618	579	550
Crude attack rates, %	29.3	17.2	19.4	20.1

a Estimated using proportional incidence ratios: the ratio of type-specific HPV infections to infections from the other nonvaccine-targeted HPV types excluding 31, 33, and 45 in vaccinated cohorts compared with the unvaccinated cohort. This was to control for the rate of nonvaccine-targeted HPV-type infections in each vaccinated cohort. CI = confidence interval; HPV = human papillomavirus.

There was no difference in efficacy between 1 dose or more on persistent HPV 16 and 18 infections (Table 6). Vaccine efficacy against HPV 16 and 18 (adjusted for using the number of infections of HPV types other than 16, 18, 6, 11, 31, 33, and 45) was 92.0% (95% CI = 87.0% to 95.0%), 94.8% (95% CI = 90.0% to 97.3%), and 95.3% (95% CI = 90.9% to 97.5%) for 1, 2, and 3 doses, respectively. Differences in vaccine efficacy estimates were not statistically significant between the 1 and 3 doses (vaccine efficacy difference = 3.3, 95% CI = −6.8 to 13.5) and 2 doses (vaccine efficacy difference = 2.8, 95% CI = 7.7 to 13.4). Vaccine efficacy against all 4 vaccine-targeted HPV types was 87.0% (95% CI = 81.2% to 91.0%) for 1 dose, 88.0% (95% CI = 81.6% to 92.2%) for 2 doses, and 86.9% (95% CI = 80.6% to 91.2%) for 3 doses. Estimates for cross-protective efficacy against persistent HPV 31, 33, and 45 after 1, 2, and 3 doses were 29.5% (95% CI = 4.9% to 47.8%), 3.6% (95% CI = -28.9% to 28.0%), and 22.2% (95% CI = -5.4% to 42.5%), respectively. Efficacy estimates against vaccine-targeted and cross-protective persistent HPV types (16, 18, 6, 11, 31, 33, and/or 45) were 63.7% (95% CI = 53.2% to 71.8%), 55.9% (95% CI = 43.0% to 65.8%), and 67.4% (95% CI = 56.8% to 75.4%) for the 1, 2, and 3 doses, respectively.

**Table 6.** Vaccine efficacy against persistent HPV infections

	Unvaccinated	1 dose	2 doses (days 1 and ≥180)	3 doses (days 1, 60, and ≥180)
Women assessed, No.	1273	3022	2311	2172
Persistent HPV 16 and/or 18 infections				
Observed events	35	4	2	2
Crude attack rates, %	2.7	0.1	0.1	0.1
Vaccine efficacy,^a^ %		92.0	94.8	95.3
95% CI		(87.0 to 95.0)	(90.0 to 97.3)	(90.9 to 97.5)
Difference in vaccine efficacy				
Alternate dose, 1 dose, %			2.8	3.3
95% CI			(-7.7 to 13.4)	(-6.8 to 13.5)
Persistent HPV 6 and/or 11 infections				
Observed events	3	3	3	4
Crude attack rates, %	0.2	0.1	0.1	0.2
Vaccine efficacy,^a^ %		29.6	9.0	−10.2
95% CI		(-45.1 to 65.7)	(-87.1 to 55.7)	(-117.4 to 44.1)
Difference in vaccine efficacy				
Alternate dose, 1 dose, %			−20.6	−39.8
95% CI			(-150.8 to 109.6)	(-178.8 to 99.2)
Persistent HPV 16, 18, 6 and/or 11 infections				
Observed events	38	7	5	6
Crude attack rates, %	3.0	0.2	0.2	0.3
Vaccine efficacy,^a^ %		87.0	88.0	86.9
95% CI		(81.2 to 91.0)	(81.6 to 92.2)	(80.6 to 91.2)
Difference in vaccine efficacy				
Alternate dose, 1 dose, %			1.0	−0.1
95% CI			(-12.8 to 14.8)	(-13.8 to 13.7)
Persistent HPV types 31, 33, and/or 45 infections				
Observed events	17	17	18	16
Crude attack rates, %	1.3	0.6	0.8	0.7
Vaccine efficacy,^a^ %		29.6	3.6	22.2
95% CI		(4.9 to 47.8)	(-28.9 to 28.0)	(-5.4 to 42.5)
Difference in vaccine efficacy				
Alternate dose, 1 dose, %			−25.9	−7.4
95% CI			(-77.5 to 25.6)	(-54.1 to 39.3)
Any persistent HPV infection				
Observed events	114	132	105	111
Crude attack rates, %	9.0	4.4	4.5	5.1
Vaccine efficacy,^a^ %		18.4	16.2	19.5
95% CI		(12.8 to 23.6)	(10.0 to 21.9)	(13.9 to 24.7)
Difference in vaccine efficacy				
Alternate dose, 1 dose, %			−2.3	1.1
95% CI			(-15.9 to 11.5)	(-12.3 to 14.5)
Persistent infection from high-risk HPV types (16, 18, 31, 33, 35, 39, 45, 51, 52, 56, 58, 59, 66, 68) included in the screening tests				
Observed events	100	105	87	97
Crude attack rates, %	7.9	3.5	3.8	4.5
Vaccine efficacy,^a^ %		26.0	20.8	19.8
95% CI		(23.4 to 28.5)	(17.5 to 24.0)	(16.3 to 23.2)
Difference in vaccine efficacy				
Alternate dose, 1 dose, %			−5.2	−6.2
95% CI			(-21.1 to 10.6)	(-21.7 to 9.2)
Persistent HPV 16 infections				
Observed events	25	1	0	0
Crude attack rates, %	2.0	0.0	0.0	0.0
Vaccine efficacy,^a^ %		97.2	100.0	100.0
95% CI		(93.2 to 98.8)	–	–
Difference in vaccine efficacy				
Alternate dose, 1 dose, %			2.8	2.8
95% CI			(-6.5 to 8.5)	(-6.0 to 8.3)
Persistent HPV 18 infections				
Observed events	11	3	2	2
Crude attack rates, %	0.9	0.1	0.1	0.1
Vaccine efficacy,^a^ %		80.8	83.5	85.0
95% CI		(65.4 to 89.3)	(67.2 to 91.6)	(70.2 to 92.4)
Difference in vaccine efficacy				
Alternate dose, 1 dose, %			2.7	4.2
95% CI			(-28.6 to 33.9)	(-25.7 to 34.0)
Persistent HPV 31 infections				
Observed events	7	9	5	4
Crude attack rates, %	0.5	0.3	0.2	0.2
Vaccine efficacy,^a^ %		9.4	35.0	52.8
95% CI		(-43.1 to 42.6)	(-10.3 to 61.7)	(16.8 to 73.2)
Difference in vaccine efficacy				
Alternate dose, 1 dose, %			25.6	43.3
95% CI			(-55.8 to 106.9)	(-35.6 to 122.2)
Persistent HPV types 33 infections				
Observed events	7	6	7	6
Crude attack rates, %	0.5	0.2	0.3	0.3
Vaccine efficacy,^a^ %		39.6	9.0	29.2
95% CI		(0.0 to 63.5)	(-47.4 to 43.8)	(-17.2 to 57.1)
Difference in vaccine efficacy				
Alternate dose, 1 dose, %			−30.6	−10.5
95% CI			(-113.5 to 52.3)	(-83.2 to 62.2)
Persistent HPV types 45 infections				
Observed events	4	2	6	6
Crude attack rates, %	0.3	0.1	0.3	0.3
Vaccine efficacy,^a^ %		64.8	−36.5	−24.0
95% CI		(24.7 to 83.5)	(-143.4 to 23.4)	(-121.2 to 30.5)
Difference in vaccine efficacy				
Alternate dose, 1 dose, %			−101.3	−88.8
95% CI			(-252.4 to 49.8)	(-225.8 to 48.2)
Other persistent nonvaccine-targeted HPV types excluding 31, 33 and 45 infections				
Observed events	81	115	89	98
Crude attack rates, %	6.4	3.8	3.9	4.5

a Estimated using proportional incidence ratios: the ratio of type-specific HPV infections to infections from the other nonvaccine-targeted HPV types excluding 31, 33, and 45 in vaccinated cohorts compared with the unvaccinated cohort. This was to control (standardize) for the rate of nonvaccine-targeted HPV-type infections in each vaccinated cohort. CI = confidence interval; HPV = human papillomavirus.

## Discussion

This IARC study has periodically reported on immunogenicity and compared the efficacy of a 1, 2, and 3 doses of Gardasil since the study was initiated 15 years ago (9,13). Our previous publication in 2021 reported 95.4% (95% CI = 85.0% to 99.9%) efficacy of a single dose against persistent HPV 16 and 18 infections at a median follow-up of 9 years based on the evaluation of 2135 single-dose recipients (9). It is reassuring to observe no waning of efficacy over a longer follow-up period (over median 12 years) among a larger number of single-dose recipients (n = 3022) assessed for persistent infections. Earlier, we reported only 1 breakthrough persistent HPV 16 infection (despite vaccination) in the single-dose cohort. HPV 16 persistent infections have not been detected since then, and subsequent cervical sample analysis showed that the breakthrough persistent infection had cleared. High protection from a single dose is confirmed by almost zero prevalence of HPV 16 observed in the single-dose recipients undergoing screening, even though the genotyping test used for screening was different from the test used to estimate vaccine efficacy.

Few HPV 18 persistent infections were detected in each of the different dose cohorts. Protection observed against persistent HPV 18 is lower, and this is probably because of the small number of infections detected in vaccinated and unvaccinated cohorts.

High single-dose efficacy has been demonstrated in vaccines of different valency. While our study provides evidence supporting the high protective efficacy of a single dose of quadrivalent vaccine against persistent HPV 16 and 18 infection, a Kenyan randomized controlled trial has shown similar results using a bivalent vaccine (97.5%, 95% CI = 90.0% to 99.4%) and a nonavalent vaccine (98.8%, 95% CI = 91.3% to 99.8%) (14). Consistency in the results across various trials reinforces the high efficacy of a single dose of HPV vaccine.

Prevention of persistent HPV 16 and 18 infections is a robust surrogate outcome for protection against cervical cancer (15). The natural history of HPV 16 and 18 persistent infection was documented in a Danish cohort study among women with normal cervical cytology at baseline. They observed that 26.7% (95% CI = 21.1% to 31.8%) of women with persistent HPV 16 developed CIN 3 or worse within 12 years of follow-up (16). The proportion was 19.1% (95% CI = 10.4% to 27.3%) for women with persistent HPV 18. Thus, the high efficacy observed for a single dose of the vaccine to protect against HPV 16 and 18 will result in substantial prevention of CIN 3 or cervical cancer.

With lasting efficacy exceeding 90% against persistent HPV 16 and 18, the impact of a single dose on cervical cancer prevention would be substantial in low- and middle-income countries. A recent systematic review at IARC observed that 71.5% of cervical cancers in Africa and 82.7% in central, western, and southern Asia are attributed to these 2 HPV types (17). As observed consistently in our study and other studies, the cross-protective efficacy against persistent HPV types phylogenetically closely related to HPV 16 (HPV 31 and 33) or HPV 18 (HPV 45) is generally low with Gardasil* (18). Analysis of data from a phase III randomized controlled trial demonstrated moderate efficacy (46%) for 3 doses of Gardasil only against HPV 31 persistent infection in females aged 16-26 years (19). We observed a small cross-protective efficacy of a single dose (29.6% against persistent HPV 31, 33, and 45 infections) with a wide confidence interval (95% CI = 4.9% to 47.8%).

The present study demonstrates how vaccination will impact HPV positivity and requirements for colposcopy within cervical screening programs in real-life settings. Prevention of HPV 16 and 18 infections will lead to substantial reduction in HPV positivity, the number of women requiring colposcopy, and the overall number of women with low- and high-grade lesions. Nevertheless, a substantial number of high-risk HPV types not targeted by the vaccines will still be detected through screening tests. Because the risk of additional types causing CIN 3 or cervical cancer is very low, an appropriate triage test for these women would be necessary to reduce referrals to colposcopy (20). Though the numbers are still small, absence of a single case of HPV 16- and 18-associated CIN 2 or worse in our screened cohort is encouraging. The only other study that has reported long-term vaccine efficacy of a single dose (at 11 years postvaccination) against persistent HPV 16 and 18 infection is the Costa Rica Vaccine Trial. The single-dose group in the Costa Rica Vaccine Trial was too small to investigate efficacy against the CIN 2 or worse endpoint (21). The IARC trial is the only study to date with a sufficient sample size of screening-eligible women to investigate cervical precancer as an endpoint. Further follow-up of vaccinated and unvaccinated cohorts in the trial will demonstrate durability of protection against the CIN 2 or worse endpoint.

Our study has a few limitations, the most significant of which is the possibility of a selection bias because of its nonrandomized design. However, given that the cohorts were spontaneously created among randomly allocated participants because of the abrupt interruption of vaccination and that neither the participants nor the investigators had any control over cohort allocations, the possibility of any selection bias has been minimized. A comparative analysis (reported in our earlier publication) of the age and sociodemographic characteristics of the cohorts at baseline demonstrated minimal differences that were adjusted during the vaccine efficacy analysis (10).

Another limitation is that the unvaccinated cohort selected post hoc—who had a substantially higher proportion of nonvaccine-targeted HPV types—had a higher risk of getting HPV infections compared with the vaccinated cohorts. To control for the imbalance, we estimated vaccine efficacy based on the odds ratio obtained when the proportion of type-specific HPV infections from HPV types other than 16, 18, 6, 11, 31, 33, and 45 in the vaccinated cohorts was divided with the similar proportion in the unvaccinated cohorts as suggested by Peter Sasieni (11). Extremely low rates of persistent HPV 16 and 18 infections detected for a single-dose or higher gives us confidence in our vaccine efficacy estimates, despite this limitation.

The use of those other HPV types (ie, types other than 16, 18, 6, 11, 31, 33, and 45) to control for the imbalance also resulted in unexpected vaccine efficacy estimates for incident HPV 6 and 11 infections (1 dose: -59.7%; 2 doses: -59.4%; 3 doses: -49.5%). The higher proportionate incidence estimates in the vaccinated cohorts compared with the unvaccinated group brought the vaccine efficacy to at least -50% for HPV 6 and 11. A detailed explanation on how we arrived at these vaccine efficacy estimates is given in the Supplementary Material (available online). This phenomenon was observed in the vaccine efficacy estimation of HPV 6 and 11 as the number of incident HPV 11 infections was quite low compared with the number of incident other HPV infections in the unvaccinated group (Supplementary Table 2, available online).

As mentioned above, detection of HPV 11 in this study was low in the vaccinated and unvaccinated cohorts. In the absence of large population-based estimates for low-risk HPV types, this could well be reflective of the overall low HPV 11 rates in this population. Another possibility could be failure of the HPV genotyping assay employed in this study to be able to detect the virus. This is highly unlikely, as the assay has consistently qualified WHO LabNet testing for quality control multiple times since 2018 and found to be proficient at every instance. Each round of testing included blinded HPV 11 samples in low and high concentrations (50 and 500 genome equivalents) as single type and in combination with other HPV types. In every proficiency round, such samples were correctly identified thus indicating no loss of detection at the assay level.

This study has shown convincing evidence for the long-term efficacy of a single dose of HPV vaccination. Using 2 independent genotyping technologies (Luminex and HPV screening tests) performed in laboratories completely blinded to dose allocations, this IARC study confirms the high protective efficacy of a single dose against persistent HPV 16 and 18 infections. The cohort size in our study to assess single-dose efficacy is the largest in any trial reported to date (22). This ongoing study will continue to generate evidence on longer-term efficacy and immunogenicity so that national and international stakeholders can recommend a single dose of the vaccine with greater confidence. An increasing number of HPV vaccine manufacturers are obtaining market authorization and WHO prequalification. More evidence needs to be generated on the single-dose efficacy of new vaccines through immunobridging studies. This will ensure that all countries have an adequate and affordable vaccine supply to expand target age and opt for gender-neutral vaccination thus making the programs more efficient, resilient, and impactful and accelerating progress toward cervical cancer elimination.

## Supplementary Material

lgae042_Supplementary_Data

## Data Availability

External researchers can make written requests to the IARC for sharing of data after publication. Requests will be assessed on a case-by-case basis in consultation with lead and co-investigators. A brief analysis plan and data request will be required and reviewed by the investigators for approval of data sharing. When requests are approved, anonymized data will be sent electronically in password-protected files. All data sharing will abide by rules and policies defined by the sponsor; relevant institutional review boards; and local, state, and federal laws and regulations. Data sharing mechanisms will ensure that the rights and privacy of individuals participating in research will be protected at all times.
