# Supplementary material for: A prospective cohort study comparing efficacy of 1 dose of quadrivalent human papillomavirus vaccine to 2 and 3 doses at an average follow up of 12 years postvaccination
Source: J Natl Cancer Inst Monogr. 2024 Nov 12;2024(67):317–28. doi: 10.1093/jncimonographs/lgae042 (PMC11555276; doi:10.1093/jncimonographs/lgae042)
Supplement: lgae042_Supplementary_Data [file lgae042_supplementary_data.docx]

**Supplementary material**

**Detailed statistical methods**

The profile of participants providing samples for HPV genotyping included assessment of data for women eligible for sample collection for HPV genotyping, women with at least one sample analysed, women eligible for the second sample collection among those whose first sample was analysed, women with at least a second sample analysed, age (in years) at first sample collection, and times (in years) between first vaccination dose and last sample collection, marriage and first cervical cell sample collection, and between consecutive sample collections.

Women were eligible for cervical cell sample collection 18 months after marriage or 6 months after first childbirth, whichever was first. The cervical screening participants profile included assessment of data for women eligible for screening, women screened at least once, screen negative women undergoing second round of screening, age at first screen and time between first vaccination dose and first screen. Women were eligible for screening on turning 25 years. All categorical variables of the profile of participants providing samples for HPV genotyping and screening, and estimation of incident and incident persistent infections based on HPV genotyping were presented as proportions. The distribution of profile continuous variables was shown as median together with their interquartile ranges.

*Study outcomes*

The primary outcome assessed in this analysis was persistent HPV 16 and/or18 infection (HPV 16/18). We looked at incident HPV 16/18 infections, and HPV 16/18-related CIN 2 or worse lesions as secondary outcomes; and as exploratory outcomes, persistent and incident infections from HPV6/11, HPV 31/33/45, oncogenic high-risk HPV 16/18/31/33/35/39/45/51/52/56/58/59/66,68) infections included in the screening tests and any HPV type.

An HPV infection was defined as incident if it were detected in any participant sample, and as persistent if detected in two consecutive samples collected at least 10 months apart. Each participant was counted once in the analysis. Women with multiple incidence or multiple persistent infections of a particular outcome were counted only at the first instance of that outcome. Both HPV outcomes were assessed using the Luminex assay.

*Statistical analysis*

Vaccine efficacies in prevention against the HPV incidence and persistent study outcomes were assessed using proportional incidence ratios method as explained by Sasieni P^1^. In this method, the vaccine efficacy estimates were obtained in two steps: a) obtaining the proportion of the number of HPV infections for a particular outcome out of the number of non-vaccine targeted HPV infections excluding types 31, 33 and 45 (i.e., HPV types other than 6, 11, 16, 18, 31, 33, and 45). b) obtaining the proportionate ratio as the proportion for each vaccinated cohort divided by the proportion for the unvaccinated cohort. In this method, the vaccine efficacy estimates for each vaccinated cohort are controlled for rate of non-vaccine targeted HPV infections. This method was used after observing that the proportion of non-vaccine targeted HPV types is reasonably homogeneous, even between study sites whose prevalence of HPV infections is very different (supplementary table 1 below). The vaccine efficacy estimate for each vaccinated cohort was then calculated as (1-proportionate ratio) x 100. The 95%CI confidence intervals of the vaccine efficacy estimates were obtained using the adjusted inverse hyperbolic sine intervals derived by Fagerland and Newcombe^2^. The difference between the vaccine efficacy estimates (together with their likelihood-based 95% CIs) of the single and the other two alternative dose groups were done using methods developed by Sampson and Gail^3^. If the 95% CIs of the estimates difference include zero, then the vaccine efficacies of the two groups compared were deemed not significantly different.

**Detailed pathway on how the unexpected VE estimates of incident HPV 6/11 infections were arrived at**

The proportionate incidence estimates obtained by dividing the number of incident HPV 6/11 by the number of the other incident non-vaccine targeted HPV types (i.e., types other than HPV 16, 18, 6, 11, 31, 33 and 45) obtained separately for each dose group is consistently higher in vaccinated groups compared to the unvaccinated cohort. Especially, for incident HPV 11, these proportionate incidence estimates that we used to obtain the VE are almost 3 folds (at least 1.49 folds when HPV 6 and 11 are combined) in the vaccinated groups compared to the unvaccinated group (supplementary table 2). For example, for the single-dose group, the proportionate incidence for incident HPV 6/11 infections was 0.30 (186/618) and in the unvaccinated group 0.19 (82/435). Dividing the two proportionate incident estimates resulted in a proportionate ratio of 1.59 (0.3/0.19) that is used to obtain the vaccine efficacy of -59.1% (=[1 - 1.59]*100). Similar estimates were observed in the 3-dose and 2-dose groups. This phenomenon was observed in the VE estimation of HPV 6/11 as the number of incident HPV 11 infections was quite low compared to the number of incident other HPV infections in the unvaccinated group.

**Supplementary table 1: Study site-specific incidence of non-vaccine-targeted HPV infections excluding 31, 33 and 45 among the vaccinated and unvaccinated groups**

| **Study site** | **Vaccinated groups** | | | | | |  | | **Unvaccinated group** | | | | | |
| --- | --- | --- | --- | --- | --- | --- | --- | --- | --- | --- | --- | --- | --- | --- |
|  | **Women** | **Incidence** | | **Incidence based** | |  | | **Women** | | **Incidence** | | **Incidence based** | |  |
|  | **assessed** | **(overall)** | | **on first sample** | |  | | **assessed** | | **(overall)** | | **on first sample** | |  |
|  |  |  | | **collections** | |  | |  | |  | | **collections** | |  |
|  | **n** | **n (%)** | | **n (%)** | |  | | **n** | | **n (%)** |  | **n (%)** |  |  |
| Ambillikai | 1,891 | 412 | (21.8) | 211 | (11.2) |  | | 200 | | 60 | (30.0) | 25 | (12.5) |  |
| Barshi | 5,757 | 824 | (14.3) | 397 | (6.9) |  | | 188 | | 34 | (18.1) | 15 | (8.0) |  |
| Delhi | 421 | 118 | (28.0) | 53 | (12.6) |  | | 200 | | 52 | (26.0) | 21 | (10.5) |  |
| Ahmedabad | 633 | 132 | (20.9) | 63 | (10.0) |  | | 50 | | 12 | (24.0) | 3 | (6.0) |  |
| Hyderabad | 319 | 74 | (23.2) | 36 | (11.3) |  | | 248 | | 50 | (20.2) | 34 | (13.7) |  |
| Mumbai | 120 | 22 | (18.3) | 18 | (15.0) |  | |  | |  |  |  |  |  |
| Pune | 2,459 | 358 | (14.6) | 171 | (7.0) |  | | 400 | | 91 | (22.8) | 35 | (8.8) |  |
| Sikkim | 138 | 79 | (57.2) | 29 | (21.0) |  | | 100 | | 63 | (63.0) | 9 | (9.0) |  |
| Mizoram | 170 | 98 | (57.6) | 56 | (32.9) |  | | 100 | | 73 | (73.0) | 31 | (31.0) |  |
| Total | 11,908 | 2,117 | (17.8) | 1,034 | (8.7) |  | | 1,486 | | 435 | (29.3) | 173 | (11.6) |  |

**Supplementary table 2: Detailed pathway for calculation of the vaccine efficacy for incident HPV types 6 and/or 11**

| **Vaccine** | **No. of** | **No. of** | **Proportionate** | **Proportionate** | **Vaccine** |
| --- | --- | --- | --- | --- | --- |
| **dose group** | **infections** | **non-vaccine** | **incidence** | **incidence** | **efficacy** |
|  | **for a** | **targeted** |  | **ratio** | **estimate** |
|  | **particular** | **incident HPV** |  |  |  |
|  | **outcome** | **excluding** |  |  |  |
|  |  | **31, 33 and 45** |  |  |  |
| ***HPV 6 and/or 11 incident infections outcome*** |  |  |  |  |  |
| Unvaccinated | 82 | 435 | 0.19 |  |  |
| Three-dose | 155 | 550 | 0.28 | 1.49 | -49.0 |
| Two-dose | 174 | 579 | 0.30 | 1.59 | -58.8 |
| Single-dose | 186 | 618 | 0.30 | 1.59 | -59.1 |
| ***HPV 6 incident infections outcome*** |  |  |  |  |  |
| Unvaccinated | 49 | 435 | 0.11 |  |  |
| Three-dose | 31 | 550 | 0.06 | 0.50 | 49.6 |
| Two-dose | 28 | 579 | 0.05 | 0.43 | 56.6 |
| Single-dose | 34 | 618 | 0.06 | 0.49 | 50.8 |
| ***HPV 11 incident infections outcome*** |  |  |  |  |  |
| Unvaccinated | 37 | 435 | 0.09 |  |  |
| Three-dose | 129 | 550 | 0.24 | 2.72 | -172.4 |
| Two-dose | 148 | 579 | 0.26 | 2.97 | -196.7 |
| Single-dose | 155 | 618 | 0.25 | 2.91 | -191.1 |
|  | | | | | |

**References**

1. Sasieni P. Alternative analysis of the data from a HPV vaccine study in India. *Lancet Oncol*. 2022;23(1):e9. doi: 10.1016/S1470-2045(21)00661-6.
2. Fagerland MW, Newcombe RG. Confidence intervals for odds ratio and relative risk based on the inverse hyperbolic sine transformation. *Stat Med* 2013; 32(16): 2823-36.
3. Sampson JN, Gail MH. Confidence intervals for the difference between two relative risks. *Stat Methods Med Res* 2020; 29(10): 3048-58. doi: 10.1177/0962280220915737
